# Label-Free Optical Differentiation of Single Diffusing Amino Acids at Picomolar Concentrations

**DOI:** 10.21203/rs.3.rs-9181809/v1

**Published:** 2026-03-30

**Authors:** Randall Goldsmith, Julia Rasch, Anna Clayborn, Daniel Sole-Barber, Sushu Wan, Carlos Saavedra, Alex Fairhall, Frank Hu, Jeffrey Bartz, Yulia Podorova, Anders Bergsten, Z. Donte’ Young, Itzel Ruiz Barrera, T H Kendrixe Mone, Nasrin Asgari, Thomas Markland

**Affiliations:** University of Wisconsin-Madison; University of Wisconsin - Madison; University of Wisconsin - Madison; University of Wisconsin – Madison; University of Wisconsin-Madison; University of Wisconsin - Madison; University of Wisconsin-Madison; Stanford University; University of Wisconsin - Madison; University of Wisconsin - Madison; University of Wisconsin - Madison; University of Wisconsin - Madison; University of Wisconsin - Madison; University of Wisconsin - Madison; University of Wisconsin - Madison; Stanford University

## Abstract

Label-free single-molecule detection schemes can provide key molecular information. Here, we deploy the elevated light-matter interaction in locked high-finesse fiber-based Fabry-Pérot microcavities (FFPCs) to push label-free detection to the single-amino acid limit, even to the smallest amino acid, glycine. Varying the sensitivity of the locked FFPC allows differentiation of subsets of amino acids at the single-molecule level, with examples including the differentiation of a small peptide from tryptophan, tryptophan from histidine, histidine from alanine, and phosphorylated threonine from threonine. Molecular detection events are visible as neuron-like signal bursts of decreased FFPC transmission, which can be replicated by simulations that include non-linear coupling between optical and thermal parameters of the locked FFPC. Importantly, differentiation was achieved in solution at pM concentrations, making the detection process suitable for integration with single-molecule processive-protein degradation. Thus, this combination of sensitivity and differentiating ability makes FFPCs a promising new photonic technology element for protein sequencing.

Amino acids are the fundamental subunit of proteins and are involved in cell signaling, regulation of gene expression, and metabolism^1^. Due to post translational modifications (PTMs) and alternate RNA splicing, the complexity of the proteome substantially exceeds the transcriptome alone^2^. There is no method to amplify proteins as there is for nucleic acids^3^. Further, bulk protein sequencing techniques like Edman degradation require multiple fluid handling steps reducing throughput, are limited to homogenous samples and peptides less than 50 amino acids, and often cannot handle PTMs^3,4^. Such bulk techniques, as well as sequencing via mass spectrometry, are difficult to apply when copy number is low^4^. Given these technical challenges and the massive societal interest due to the connection between the proteome and disease etiology, there is substantial interest in new protein sequencing technologies. Single-molecule techniques, which thrive in molecule-by-molecule sequencing operation, can address these issues by employing fast and highly processive enzymes, as has been accomplished with DNA sequencing, but identifying protein sequence at the single-molecule level remains a challenge. Here, we present a fundamentally new optical approach for the label-free detection and differentiation of single amino acids that is potentially compatible with processive enzyme degradation.

Single-molecule sequencing has been pursued via multiple approaches.^5,6^ Fluorescence-based single-molecule protein sequencing has shown promise^7–9^, but ultimately struggles due to both the unique chemistry needed to label all twenty amino acids along with the need for 20 unique fluorophores without crosstalk^3^. On the other hand, label-free techniques offer an increasingly desirable way forward. Spectroscopic identification of all twenty amino acids has been accomplished via surface-enhanced Raman scattering (SERS)^10,11^, but required adsorption of an amino acid monolayer on plasmonic nanoparticles. Another class of methods relies on tunneling currents through single molecules passing through molecular junctions. Milestones include distinguishing between a subset of amino acids and a post translational modification (PTM)^12^ and discriminating four amino acids and their enantiomers^13^. Laser tweezers and solid-state nanopores also offer promising single-molecule readouts.^6,14–16^ Building on the success and commercialization for DNA sequencing, label-free protein sequencing via biological nanopores is the closest to technological maturity^17^. In one advancement, the ability to discern all twenty amino acids along with some PTMs was demonstrated^18^. However, most of these experiments are performed at mM amino acid concentrations. For a method to be compatible with fast and potentially multiplexed single-enzyme-based sequencing approaches, the identification of the enzyme-released amino acid must occur quickly and at low or sub nM concentrations. Further, amino acid identification with ion channels also suffers from an inability to perform consecutive measurements on the same analyte via passage through several nanopores in series, a process that can improve fidelity. Thus, there is still a need for new techniques that can quickly detect and distinguish single amino acids at low concentrations.

Optical label-free strategies offer an alternative path forward and have already shown the ability to detect single molecules and particles^19–38^. One methods class relies on single-molecule interferometric scattering microscopy (iSCAT)^23^, which enables quantification of molecular weight via mass photometry^25^ and detection down to 6–10 kDa with use of machine learning^39,40^. When combined with nanofluidics, the ability to measure both mass and hydrodynamic radius^26,39^ has been demonstrated. However, detection of molecules as small as amino acids (75–204 Da) has not been demonstrated. Another label-free methods class relies on dielectric optical microresonators, which due to their high finesse (F) and low mode volume (V), allow for enhanced light matter interactions^41,42^. These microresonators have been used for detection and spectroscopy of single molecules and particles^20,22,27–36,43–46^. However, these techniques are unable to detect or differentiate freely-diffusing single amino acids. Recently, we reported that open-access fiber-based Fabry-Pérot microcavities^31–34,43,47^ (FFPCs) enable detection and hydrodynamic profiling of small (down to 1.2kDa), solution-phase biomolecules in a label-free manner at low, pM concentrations^27^, as well as an underlying quantitative model^48^. Here, we report the label-free optical detection of single amino acids, even down to the smallest amino acid, glycine, using FFPCs in a rolling detection window at pM concentrations. Further, differentiation between a subset of amino acids is demonstrated, as well as the differentiation between an amino acid and its PTM version, though differentiation of the full palette of 20 amino acids at pM concentrations is still an outstanding goal. By offering a far-field (micrometer) scale detection scheme, this method, the first that does not require a near-field (nanometer) scale interaction, also offers significant advantages for integration and reproducibility. These results elevate far-field photonics-based techniques to the top tier of potential protein sequencing approaches.

## Setup and Solution-Phase Amino Acid Detection

**Figure 1:**
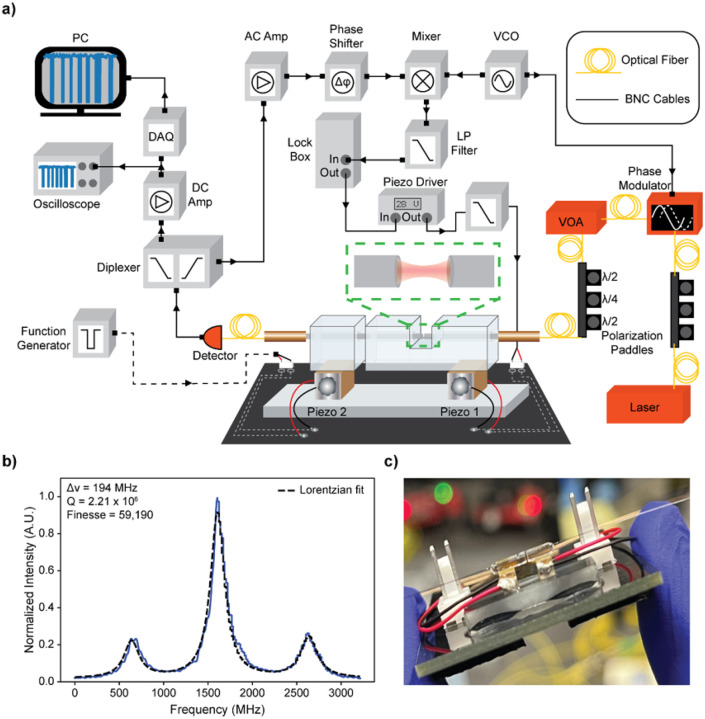
Setup and FFPC properties. a) A schematic of the setup used to detect single diffusing amino acids. A 640 or 660 nm laser (linewidth <1 MHz) was sent through polarization paddles and into a phase modulator (modulated at 200 MHz with a voltage-controlled oscillator (VCO)) and then through polarization paddles before being injected into the FFPC. The power injected into the FFPC was controlled with a voltage optical attenuator (VOA). The transmitted light was collected on an avalanche photodiode (APD). This signal was then split into low and high frequency components using a diplexer. The low frequency component was used to monitor and record the molecular signals on a DAQ. The high frequency component was mixed with the VCO output to create an error signal. This error signal was then sent to the lock box, which applied active feedback to piezoelectric one to maintain resonance. A zoom in of the FFPC which rests in the half cut of the ferrule is pictured in the green box. The full cut in the ferrule is located to the left. b) A FFPC length scan depicting an optical resonance with phasemodulated sidebands at 1 GHz. c) An image depicting the FFPC and FFPC holder system being held in a hand.

FFPCs (Figure 1) confine and store light at discrete optical wavelengths as described by the resonance condition:

(1) mλ=2nL

where m is the longitudinal mode number, *λ* is the vacuum wavelength of light injected into the FFPC, *n* is the refractive index of the medium, and *L* is distance between the two mirrors (Section S3.5). FFPCs were constructed as previously described^27,48,49^. Two optical fibers with flat cleaved, ablated, and mirror-coated faces were inserted and glued into the bore of a cut, fused silica ferrule glued atop two piezoelectric transducers. Typical FFPC parameters were length of 10 μm, radius of curvature (ROC) of 33 μm, mirror diameters of 19 μm, and a mode volume of 19 μm^3^. The finesse ranged from about 45,000 – 60,000 (Q factor = 1.8 – 2.4 × 10^6^, linewidths 190–250 MHz) in water and input power ranged from 8 – 70 μW. The optical setup (Figure 1a) includes a static pump wavelength (640 nm or 660 nm) injected into the FFPC through an optical fiber. Optical modes were probed by tuning the FFPC length via one piezo transducer (Figure 1a, “piezo 1”). FFPC transmission (Figure 1b) was collected and monitored on an avalanche photodiode (APD). The FFPC was actively maintained on resonance using Pound-Drever-Hall locking (PDH)^50^. To tune the system response towards single-amino acid detection, a function generator sent voltage pulses to a second piezo transducer (Figure 1a, “piezo 2”) to mimic molecular perturbations. For molecular detection experiments, amino acids were prepared at picomolar concentrations, and these solutions were placed in the FFPC and held in place by surface tension.

**Figure 2:**
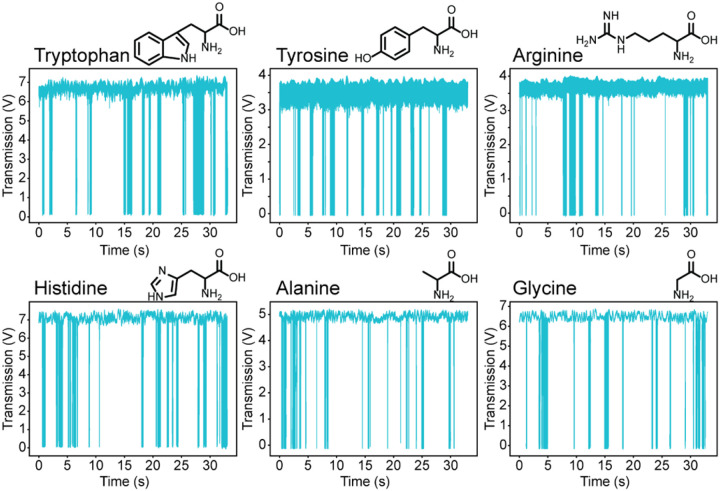
Traces showing perturbations to the locked FFPC due to single amino acids. Each drop in transmission corresponds to a signal burst (see Figure 3a) from a single amino acid diffusing through the FFPC mode volume. Each amino acid was added to the FFPC at a concentration of approximately 5 pM.

As seen in Figure 2, single amino acids interact with the FFPC mode volume and generate large drops in transmission. Detection of six amino acids is presented, ranging in size from tryptophan (204.23 Da) to glycine (74.07 Da), and all are observed with signal to noise of nearly 50:1 (Figure S6). To verify that detected signals arise from single amino acids, blank water traces were collected before and after amino acids were added to the FFPC, with little to no observation of signal peaks (Figure S8). Further, the number of detected events scales linearly with concentration (Figure S10). Following differentiation protocols discussed below, a single alanine and an alanine dimer were distinguished (Figure S9, S13), suggesting that dimer or larger aggregate impurities are not representative of the detected species. Finally, the capability to differentiate signal arising from different single amino acid species, as discussed below, strongly supports the hypothesis that signals originate from single amino acids.

## Mechanism

Signal is generated from refractive index perturbations, whereby molecules diffusing through the optical mode volume present a refractive index that is different from the bulk solvent in the FFPC. This shift in the average refractive index results in an extremely small perturbation to the FFPC, resulting in a resonance shift of less than 1 part in 10^5^ of the already narrow FFPC linewidth (Section S3.1). However, this shift results in slight (~mK) cooling of the FFPC due to the concomitant drop in coupled power, resulting in more resonance shift, and an amplifying photothermal cascade^27,48,51,52^. In this context, the propensity for a molecule to trigger an observable signal scales with its excess polarizability, *α*_*ex*_, which results in a more significant refractive index perturbation, and diffusion constant, *D*, which allows exhibition of dynamics faster than the PDH locking bandwidth and better overlap with the molecular velocity filter window^27,48^. The amplified signal from single molecules manifests as large drops in the FFPC transmission, providing superb signal to noise. However, use of the previously described signal transduction scheme^27,48^ does not allow detection of the smallest amino acids.

**Figure 3:**
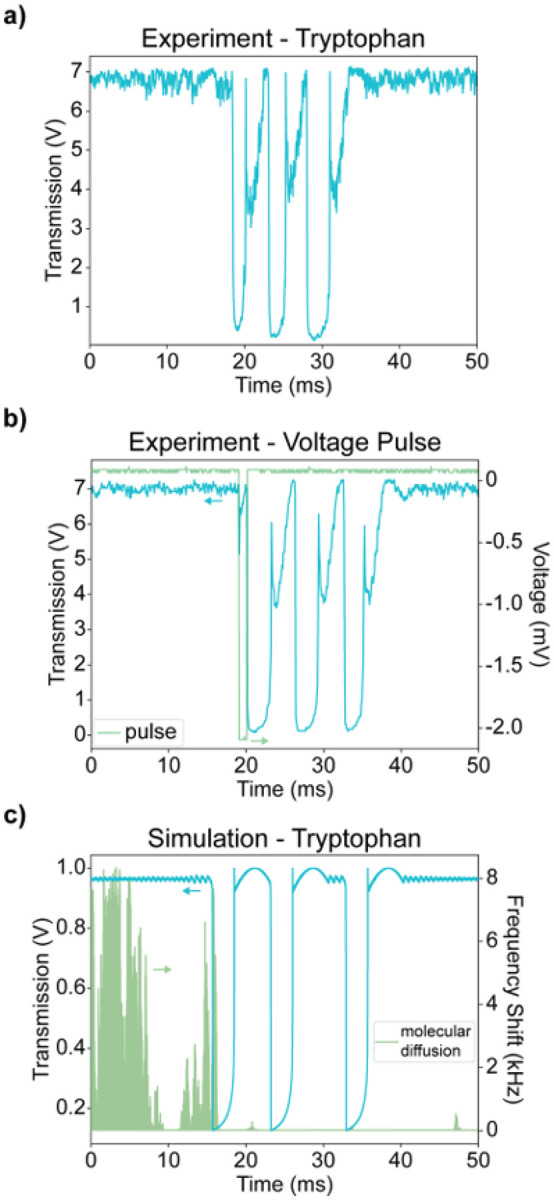
a) A transmission over time trace showing an experimental detection event from a freely diffusing tryptophan. b) A trace showing both experimental output transmission and input pulse perturbation to the FFPC. The green trace is the experimental voltage pulse applied to the FFPC, and the blue trace is the FFPC response. c) A simulated trace showing the output of the FFPC as a result of a tryptophan undergoing Brownian motion in the vicinity of the mode volume. The green trace is the FFPC resonance shift induced by the tryptophan, and the blue trace is the FFPC response. Small green/blue arrows indicate correlation between trace and y-axis in b,c).

To push the technique to enough sensitivity to detect even glycine, a new detection regime was realized with a qualitatively different output signal. This regime (Figure S1) was accessed at higher input powers, higher finesse values (Section S1.1), and lower locking bandwidth (Figure S14). In previous work^27^, single-molecule detection yielded a single inverted signal peak, where different molecular transients exhibited a range of peak heights and widths. In this new modality, a single perturbation to the locked FFPC results in a burst of consecutive drops in transmission without a return to the baseline at maximum transmission (Figure 3a), a response reminiscent of burst-firing neurons^53^. Each of the “spikes” in Figure 2 is a dense burst. The second key difference is the peaks in this detection regime are saturating, consistently dropping to a near-minimum transmission level, and exhibiting little variations in peak amplitude (Figure 3a, Figure S2). Both of these behaviors seen in single-amino acid traces can be experimentally replicated by applying a single square voltage pulse to a piezo transducer (Figure 3b). In this burst-firing regime, peak widths are governed largely by system parameters and not by molecular motion (Figure S3), precluding the possibility of direct hydrodynamic profiling^27^.

Burst-firing dynamics triggered by the Brownian motion of tryptophan can be replicated in numerical simulations that capture the coupling between optical and thermal degrees of freedom of the FFPC^48^ (Figure 3c, Section S2.1). A key input to this simulation is the *α*_*ex*_ of the amino acids. While there are a number of ways to calculate *α*_*ex,*_^54^ for such small molecules, perturbation of the aqueous solvation shell might be expected to play a major role. Building on previous calculations relying on implicit solvation^55^, we performed molecular dynamics that include explicit solvation followed by density functional theory (DFT) calculations of the full polarizability tensor (Section S2.2) for determination of *α*_*ex*_. Average values of *α*_*ex*_ were seen to increase by 25–100% relative to predictions from implicit solvent models^55^, highlighting the contribution of the oriented solvation shell to *α*_*ex*_ for such small molecules. The maximum component of *α*_*ex*_ also shows substantial and elevated deviations from the average depending on molecular orientation and instantaneous water conformation, allowing access to transiently heightened light-matter interactions (Figure S5).

## Differentiating Single Diffusing Amino Acids

**Figure 4:**
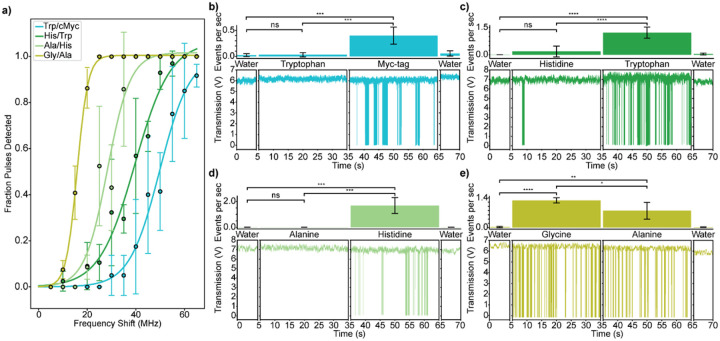
Label-free single-molecule amino acid differentiation. a) The fraction of square-wave input pulses that triggered signal in the FFPC versus amplitude of input frequency shift. Each colored trace corresponds to the two-molecule comparison experiment represented in the rest of the figure. Lines are sigmoidal fits. b-e) FFPC transmission levels (bottom) and bar graphs (top) representing the two-molecule comparison experiments. For each experiment, the bar graphs show the number of detection events per second for the water control before and after and for the two molecules being compared at 10 pM. The FFPC transmission traces below the bar graphs display example data for each experimental condition. Asterisks indicate statistical significance: ns p > 0.05, * p ≤ 0.05, ** p ≤ 0.01, *** p ≤ 0.001, **** p ≤ 0.0001

After demonstrating the ability to detect freely diffusing single amino acids, a series of experiments was designed using the new burst-firing detection regime to evaluate the possibility of distinguishing between different species of amino acid. Different amino acids possess different *α*_*ex*_ and *D* values and thus should require different minimum system sensitivities to detect. In these trials, the system was configured as a filter, with FFPC locking parameters tuned to allow for little to no detection of the smaller molecule, but sufficient sensitivity to detect the larger molecule. However, this process is complicated by FFPC-to-FFPC differences as well as drift in the properties of a single FFPC over time. Consequently, an internal calibration scheme was developed. To assess system sensitivity, the response of the locked FFPC was recorded as voltage pulses were applied to the piezo transducer of known FFPC length displacement (and thus, frequency shift), allowing for comparison across experiments. Specifically, this calibration was performed by injecting voltage pulses of varying amplitudes twice a second for a duration of 1 ms to induce a known frequency shift of the FFPC. These voltage pulses were then converted to frequency shifts (Methods). Then, the fraction of the input pulses that elicited molecule-like system responses was calculated (Figure 4a, S7)

Once the locked FFPC was internally calibrated, differentiation was attempted while maintaining the system settings. Blank water traces were collected at the beginning and end of each set of experiments. For experiments in Figure 4b–d, the smaller molecule was then added to the FFPC and little to no detection events occurred (Figure S8). Then after brief rinsing, the sample was switched out for the larger molecule, and in a successful experiment, facile detection was observed. Specific details can be found in the SI.

Four differentiation comparisons were attempted: differentiating between tryptophan and Myc-tag (10AAs, MW=1.2 kDa), histidine and tryptophan, alanine and histidine, and finally glycine and alanine. These targets are representative of the range of *α*_*ex*_ values encountered among the twenty canonical amino acids^55^. In the first experiment comparing tryptophan and Myc-tag, water was added to the FFPC and calibration pulses were applied to achieve a level of sensitivity capable of detecting Myc-tag. In this regime, calibration pulses of different amplitudes were applied, and the system response was collected (Figure 4a, teal trace). Blank water traces were then collected and little to no detection events occurred (Figure 4b, first panel). Tryptophan was then added to the FFPC at 10 pM and again, little to no events were detected (Figure 4b, second panel). Differences in the rates of detection between tryptophan and water were not statistically significant. Next, Myc-tag was added at 10 pM and the number of events detected per second increased dramatically (Figure 4b, third panel). After washing, blank water traces were again collected showing only sporadic events (Figure 4b, fourth panel), as well as calibration data from another round of voltage pulses (Figure S11). Thus, the FFPC was configured to allow differentiation between Myc-tag and tryptophan.

Comparison between histidine and tryptophan follows the same protocol but at elevated sensitivity (Figure 4a, dark green trace): histidine is not observed upon addition to the FFPC, but tryptophan is robustly detected (Figure 4c), thus histidine and tryptophan can be differentiated. Finally, the protocol was repeated at an even more sensitive configuration (see leftward shifting traces in Figure 4a), allowing differentiation between histidine and alanine (Figure 4d). The last experiment compared alanine and glycine (Figure 4e). Though the requisite sensitivity to detect glycine, the smallest amino acid, was achieved, conditions were not found whereby alanine and glycine could be differentiated, as substantial detection occurred for both molecules. In this case, somewhat more glycine events were detected than alanine, likely due to slight deviations in concentration during sample preparation.

The use of voltage pulses to calibrate the FFPCs and assess differing sensitivity from experiment to experiment drastically improves the practical usability of FFPC label-free sensing. Further, this regime utilized the tunable input locking parameters to maximize sensitivity to smaller molecules. The ability to selectively tune the FFPC’s detection regime to different size molecules illustrates the use of a rolling or tunable detection window^48^, potentially offering a route to examine more complex systems like mixtures which is particularly important when studying biological systems.

## Differentiating an Amino Acid and its PTM

**Figure 5:**
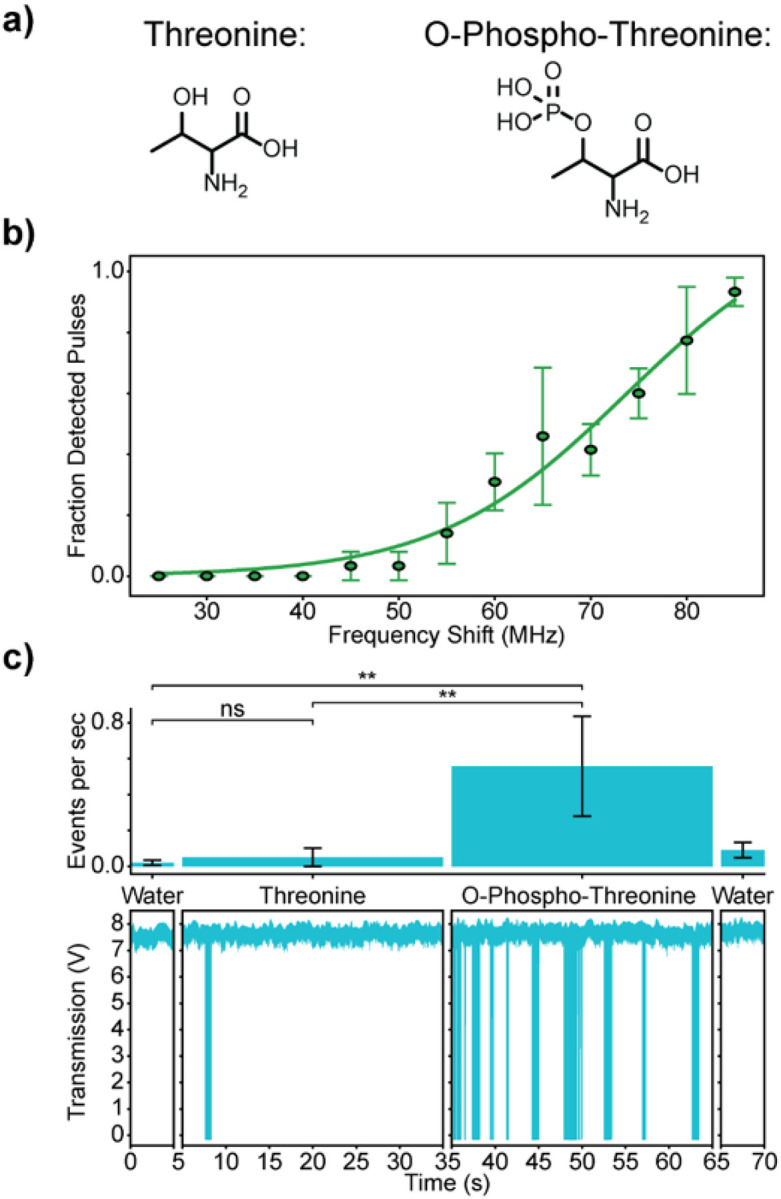
Differentiation of an amino acid and PTM version. a) Molecules being compared in the following experimental data. b) The fraction of square-wave input pulses that triggered signal in the FFPC versus amplitude of input frequency shift. c) FFPC transmission levels and bar graphs for the comparison of Threonine and phosphorylated Threonine (PTM). For this experiment, the bar graph shows the number of detection events per second for the water control before and after and for the two molecules being compared at 10 pM. The FFPC transmission traces below the bar graphs display example data for each experimental condition. Asterisks indicate statistical significance: ns p > 0.05, * p ≤ 0.05, ** p ≤ 0.01, *** p ≤ 0.001, **** p ≤ 0.0001

PTMs are chemical modifications to amino acid residues within proteins that occur after translation, creating additional complexity in the proteome, which would be entirely missed when looking exclusively at the DNA genome or RNA transcriptome. These modifications are crucial for biological processes and dysfunction in the PTM process can lead to disease^56^. Here, the ability to differentiate between a commonly modified amino acid, threonine (Thr), and its modified PTM version, O-phospho-threonine (Thr-P), was explored. The phosphorylation to threonine increases *α*_*ex*_ due to the anionic phosphate. Using the protocols described above, differentiation between Thr and Thr-P was achieved. Because of the highly polarizable phosphate group on Thr-P, the sensitivity of the FFPC was tuned (Figure 5b) to be less sensitive than the comparisons in Figure 4. After the calibration, little to no events were observed in the blank water traces (Figure 5c, panel 1). Thr was added to the FFPC and again little to no detection events occurred (Figure 5c, panel 2). Then, Thr-P was added to the FFPC and plentiful detection occurred (Figure 5c, panel 3). Finally, blank water traces were collected (Figure 5c, panel 4) as well as additional calibration data (Figure S12). This successful differentiation between an amino acid and its PTM version showcases how the sensitivity of the FFPC can be readily tuned in this detection regime to provide molecular selectivity.

## Conclusion

The ability to differentiate freely-diffusing, single amino acids is a major new capability for label-free single-molecule sensing. Distinguishing amino acids, as well as unmodified amino acids from PTMs, is the foundation of protein sequencing. In one study, a biological nanopore was able to differentiate between all 20 amino acids in a label-free manner^18^ but required high mM concentrations and high static electric fields. In another study with a different biological nanopore, detection of all twenty amino acids with limits of detection (LODs) varying from 100 nM to 1 μM concentrations was demonstrated, but differentiation of all 20 was not possible^57^. However, in both studies the LOD was defined as the minimum concentration required to detect at least 5 amino acids within 10 minutes, implying hours to sequence a single protein. Consequently, our progress toward amino acid identification at low concentration offers a potential solution to this issue. Further, biological nanopore detection, while parallelizable, does not offer an easy route for serial detection with multiple sequential nanopores. No such barrier exists here, and linear arrays of microcavities^34^ can potentially be deployed to increase precision by allowing multiple passes of the same molecule through different microcavities.

Detection of amino acids in solution is another advancement. Using plasmonic nanorods coupled to high-Q whispering gallery mode (WGM) microresonators, the detection of single aqueous ions (Zn^2+^ and Hg^2+^) has been reported^58^ with an average signal-to-noise (S/N) of ~6, a significant achievement. The mass of a Zn^2+^ ion (65.38 g/mol) is marginally smaller than glycine (75.07 g/mol). However, detection via refractive index contrast, as employed in our FFPC apparatus, was ruled out^58^ in favor of amalgamation or other surface interaction with the gold nanorod, and differentiation was not demonstrated. Thus, the direct optical single-molecule detection of solution-phase glycine with S/N~50 is a new benchmark in label-free sensing, and differentiation a major new capability.

Though all twenty amino acids cannot be differentiated due to the overlapping *α*_*ex*_^55^, our setup offers several future ways of overcoming this challenge. Linear, serial arrays of FFPCs, each with a different system responses, akin to neural heterogeneity^59^, could allow molecular identification or analysis of more complex biofluids. Measuring differences in diffusion times across known distances between microcavities would also allow the system to recover the ability to perform hydrodynamic profiling^27^, adding another orthogonal channel for distinguishing molecules. An additional method to aid in differentiation, could be the incorporation of electrodes that can allow differentiation via electrophoretic properties, as are relied on in capillary electrophoresis. It is also interesting to note that the locked FFPC possesses multiple attributes of a neuromorphic sensor^53^, including highly non-linear response deriving from tuned gain parameters, burst signal morphology, and environmental noise providing a key amplifying element akin to stochastic resonance^48^. Neuromorphic signal generation and processing may provide additional perspectives for further enhancing sensitivity, sensor robustness, or information content^60^. In total, the ability to perform label-free single-molecule detection and differentiation of amino acids offers a powerful new platform for optical sensing and identification of molecules.

## Methods

### Sample Preparation

Glycine (MilliporeSigma, 50046), alanine (MilliporeSigma, 05129), histidine (MilliporeSigma 53319), arginine (MilliporeSigma, A8094), tyrosine (MilliporeSigma, 93829) tryptophan (MilliporeSigma, 93659), threonine (MilliporeSigma, 89179) and O-Phospho-L-threonine (MilliporeSigma, 72852) samples were prepared the day of the experiment. They were dissolved to 1 mg mL^−1^ in filtered (Ø = 20 nm, Whatman Anotop, WHA68091102) ultrapure Millipore water (18 MΩ, pH 7) and diluted to the final pM concentration required for experiment. The alanine dimer (MilliporeSigma, A9502) was prepared in the same manner as the single amino acids. The Myc-tag (MilliporeSigma, M2435) sample was dissolved to 1 mg mL^−1^ in PBS (pH 7.4), aliquoted and stored at −20 °C. For experiments, aliquots were thawed on ice and diluted to the appropriate pM concentration using filtered ultrapure Millipore water. Prepared samples were kept in a chilled cooler before being analyzed.

### Piezo transducer Calibration

To convert the voltage pulses applied to the FFPC from a voltage shift to a frequency shift, the piezoelectric transducer was calibrated. A static frequency laser (640 nm or 660 nm, Hubner) was phase modulated at a known frequency (1 GHz) to apply sidebands to the resonance to serve as a frequency marker. A triangle wave was applied to the piezo being calibrated and an offset was provided by the other piezo in order to tune the FFPC length and locate the resonance. An oscilloscope (Keysight, DSOX1204G) collected the resonance with sidebands at a known frequency as the voltage ramp was applied to piezo transducer. From there, the slope of the triangle wave was fitted to get a value in volts per second. The distance between the resonance and the sidebands can be extracted in seconds and as this distance is known in frequency space it can be used to give a conversion from time to frequency. Using this information, a MHz per volt conversion factor can be calculated to convert pulse amplitudes to frequency shifts.

### Tuning FFPC Parameters

The FFPC locking parameters that were tuned to reach the unstable, burst-firing regime include the input light power, the gain of the PDH servo loop, the resistance of the low pass filter connected to piezo 1, and the set point of the PDH servo loop. Voltage pulses of similar size to the internal calibration were applied while tuning the locking parameters to identify the optimal sensitivity and will be referred to as “test pulses”. The input light power, the gain, and the resistance of the low pass filter all affect the locking bandwidth and therefore any large adjustments made to one of these locking parameters required adjustments to be made to the other locking parameters. After FFPC tuning parameters were set using the “test pulses”, the calibration was performed.

Each parameter’s role can be described separately. First, the input power was adjusted. This input power refers to the optical power injected into the FFPC (8–70 uW) and it was optimized such that the sufficient photothermal non-linearity exists to trigger the burst signal from single small molecules, but not so overly sensitive that ambient noise results in spurious burst signal. Increasing the input power into the FFPC also increases the locking bandwidth needed to stabilize the FFPC resonance and therefore adjustments of the other parameters were required. Next, the gain of the PDH servo loop was adjusted, which controls both the proportional and integrator gain of the PI controller (Vescent D2–125) or “lock box”, which controls the process by which the PI loop is able to respond to perturbations. The optimization of the gain of the PDH servo loop involved increasing the gain value if the FFPC response was too sensitive and decreasing the gain value if the FFPC response was not sensitive enough. Next, the resistance of the low pass filter, which refers to the value of the low pass filter inserted before piezo 1, was adjusted (10–32 kOhm). This low pass filter filters the output frequencies of the servo loop that are applied to piezo 1. The value of the low pass filter was optimized such that the excess electronic noise causing spurious signal was suppressed and molecular detection could occur. Increasing this value was observed to suppress spurious signal but using too high resistance values could also indirectly suppress molecular signal because it reduces the overall gain which results in a lower LBW and larger frequency detection window, which may allow for mixed molecule and ambient noise related peaks (false positives). Finally, the set point of the PDH servo loop controls the detuning between pump and center frequency that the servo loop is attempting to maintain, was optimized. This parameter was adjusted such that the signal exhibited maximum sensitivity without breaking the FFPC PDH lock. This involved moving the set point so that it was near the top of the resonance (increasing transmission) to enhance sensitivity and lowering the set point (decreasing transmission) if the PDH lock was broken. This process is then repeated until the targeted sensitivity is reached as assessed by test pulses.

### Data Collection for Single Amino Acid Experiments

At the beginning of each experiment, the same filtered (Ø = 20 nm, Whatman Anotop, WHA68091102) ultrapure Millipore water (18 MΩ, pH 7) that was used to dilute amino acid samples was added to the FFPC (8 μL). The fundamental FFPC mode was maintained and PDH locked via piezoelectric transducer length tuning of the FFPC. The FFPC locking input parameters were tuned to be able to detect the smallest possible pulses without large amounts of spurious signal. Three 33 second blank water traces were then collected, in which there was little to no signal. After, the water was mostly removed with a pipette, leaving enough water behind to keep the FFPC in water. Then, a pM solution of a single amino acid was added to the FFPC. Several 33 second traces were collected at this concentration. After the amino acid data was collected, the FFPC was cleaned by removing the amino acid solution via pipette and then ultrapure Millipore water was pipetted on the ferrule, letting it sit for a few minutes, and then the water was pipetted off the ferrule. Three 33 second traces were collected after to ensure that there was little to no spurious signal without the presence of single amino acids.

### Data collection for Differentiating Between Species

At the beginning of each experiment, the same filtered (Ø = 20 nm, Whatman Anotop, WHA68091102) ultrapure Millipore water (18 MΩ, pH 7) that was used to dilute amino acid samples was added to the FFPC (8 μL). The fundamental FFPC mode was maintained and PDH locking was achieved via FFPC piezoelectric transducer length tuning. The FFPC locking input parameters were tuned to detect pulses that corresponded to the larger of the two molecules. This correlation was determined experimentally. A series of 13 different pulse amplitudes from 5 MHz to 65 MHz, with a width of 1 millisecond and frequency of 2 Hz, were applied to piezo transducer two. Four 6-second traces for each pulse condition were collected. Next, three 33-second blank water traces were collected in which little to no spurious signal was observed. After, the water was mostly removed with a pipette, leaving enough water behind to keep the FFPC in water. Then a pM solution of the first molecule was added to the FFPC. Several 33 second traces were collected at this concentration. The FFPC was then briefly rinsed with water by pipetting water on and off the ferrule to clean. Next, a pM solution containing the second molecule was pipetted on top of the ferrule, several 33 second traces were collected at this concentration. After this data was collected, the FFPC was cleaned by removing the amino acid solution via pipette and then ultrapure Millipore water was pipetted on the ferrule, it was allowed to sit for a few minutes, and then the water was pipetted off the ferrule. Three 33 second traces were collected after to ensure that there was little to no spurious signal without the presence of single amino acids.

### Molecular Detection Data Analysis

Transmission data traces were analyzed using a custom python code to visualize and count the number of detection events over a given amount of time giving the units of events per second as seen in Figure 4 b–e. Detection events are defined to be independent clusters of peaks (drops in transmission) or single peaks that return to baseline for at least 0.5 seconds before an additional peak is detected.

### Pulse Data Analysis

The pulse data was analyzed using both the pulse trace and the transmission trace. The total number of pulses applied to the FFPC was recorded for a given input pulse amplitude, as well as the total number of detection events that corresponded to input pulses. The number of detection events versus the number of input pulses can then be calculated and is referred to as fraction pulses detected in Figure 4a. In some cases, the burst signal would oscillate for an extended period before returning to baseline, bleeding into the next input pulse. If the FFPC was oscillating before the input pulse signal, the input pulse would not be counted towards the total number of pulses (Figure S7d). In other cases, there would be a burst signal response that was not correlated with a pulse. This spurious signal was not counted as a positive FFPC response (Figure S7c).

## Supplementary Material

Supplementary Files

This is a list of supplementary files associated with this preprint. Click to download.

- SIV7submission.pdf
